# Biventricular or Conduction System Pacing for Cardiac Resynchronization Therapy: A Strategy for Cardiac Resynchronization Based on a Hybrid Approach

**DOI:** 10.3390/jcdd10040169

**Published:** 2023-04-14

**Authors:** Amato Santoro, Federico Landra, Carmine Marallo, Simone Taddeucci, Nicolò Sisti, Andrea Pica, Andrea Stefanini, Maria Cristina Tavera, Antonio Pagliaro, Claudia Baiocchi, Matteo Cameli

**Affiliations:** 1 Division of Cardiology, Azienda Ospedaliera Universitaria Senese, Viale Bracci 1, 53100 Siena, Italy; 2 Division of Cardiology, Università degli Studi di Siena, Viale Bracci 4, 53100 Siena, Italy; 3 Division of Cardiology, San Donato Hospital, Via Pietro Nenni, 52100 Arezzo, Italy

**Keywords:** cardiac resynchronization therapy, His bundle pacing, left bundle branch pacing, conduction system pacing, biventricular cardiac pacing, interventricular conduction delays

## Abstract

Background: Cardiac resynchronization therapy (CRT) is usually performed with biventricular pacing (BiVP), but recently, conduction system pacing (CSP) has been proposed as an alternative in case of BiVP failure. The aim of this study is to define an algorithm to choose between BiVP and CSP resynchronization using the interventricular conduction delays (IVCD) as a guide. Methods: Consecutive patients from January 2018 to December 2020 with an indication for CRT were prospectively enrolled in the study group (delays-guided resynchronization group, DRG). A treatment algorithm based on IVCD was used to decide whether to leave the left ventricular (LV) lead to perform BiVP or pull it out and perform CSP. Outcomes from the DRG group were compared to a historical cohort of CRT patients who underwent CRT procedures between January 2016 and December 2017 (resynchronization standard guide group, SRG). The primary endpoint was a composite of cardiovascular mortality, heart failure (HF) hospitalization, or HF event at 1 year after the date of intervention. Results: The study population consisted of 292 patients, of which 160 (54.8%) were in the DRG and 132 (45.2%) in the SRG. In the DRG, 41 of 160 patients underwent CSP based on the treatment algorithm (25.6%). The primary endpoint was significantly higher in the SRG (48/132, 36.4%) compared to the DRG (35/160, 21.8%) (hazard ratio (HR): 1.72; 95% confidence interval (CI): 1.12–2.65; *p* = 0.013). Conclusions: A treatment algorithm based on IVCD shifted one patient out of every four from BiVP to CSP, with consequent reduction in the primary endpoint after implantation. Therefore, its application could be useful to determine whether to perform BiVP or CSP.

## 1. Introduction

Cardiac resynchronization therapy (CRT) reduces morbidity and mortality in patients with heart failure (HF) and electrical desynchrony [1,2,3]. CRT is usually performed by positioning a left pacing lead into a lateral or posterolateral tributary vein of the coronary sinus. According to the latest ESC guidelines [4], CRT is indicated in symptomatic HF patients with reduced ejection function (EF, <35%) and a QRS duration ≥ 130 ms. CRT allows for interventricular and atrioventricular resynchronization, causing a reverse remodeling process associated with an improvement in functional class and systolic function [3,5]. CRT response varies from 60% to 70% and is influenced by sex, baseline QRS duration, QRS morphology, body surface area (BSA), and HF etiology [6,7,8,9,10]. Although biventricular pacing (BiVP) has long been the standard method for CRT, conduction system pacing (CSP) has recently been suggested as a reliable alternative when BiVP fails to restore the normal electromechanical activation of the heart [11]. Recent ESC guidelines suggest CSP in patients who have a high-degree AV block, underwent AV node ablation and have an ejection fraction (EF) < 50%, or in situations where technical issues arise during CRT implantation (i.e., unsuccessful coronary sinus lead implantation) [4]. However, a clear strategy to determine when CSP should be used over conventional BiVP during CRT therapy is not available yet. The aim of this study is to assess the clinical benefit of a treatment algorithm based on interventricular conduction delays (IVCD) to choose between BiVP and CSP in candidates for CRT.

## 2. Materials and Methods

### 2.1. Study Design

In this single center study, consecutive patients presenting at our institution (Azienda Ospedaliera Universitaria Senese, Siena University Hospital, Siena, Italy) from January 2018 to December 2020 with an indication for CRT were prospectively enrolled in the study group (delays-guided resynchronization group, DRG). Indications for CRT were: (1) symptomatic HF with reduced ejection fraction (EF, <35%) despite optimal medical therapy (OMT) with a QRS width >130 ms for patients with left bundle branch block (LBBB), or >150 ms for patients with right bundle branch block (RBBB); (2) high-degree atrioventricular block (AVB) associated with reduced EF (<45%); (3) AV junction ablation for rate control therapy of atrial fibrillation (AF) associated with reduced EF (<45%). A historical cohort of patients who underwent CRT at our institution from January 2016 to December 2017 was used as a control group (standard resynchronization group, SRG). Exclusion criteria in both groups were: AF with ventricular pacing (VP) < 95%; frequent premature ventricular complexes (PVC) with VP < 95%; left ventricular (LV) lead dislodgment; follow-up not available. This study was performed in accordance with the Declaration of Helsinki and was reviewed and approved by the local ethics committee.

### 2.2. Clinical Data

Baseline demographic, clinical, eletrocardiographic, and echocardiographic data of included patients were prospectively collected and reported in a dedicated database. The same data were collected at the 1-year follow-up as well. All echocardiographic examinations were performed by experienced operators blinded to procedural aspects and clinical outcomes using the GE Vivid iQ ultrasound system equipped with an adult transthoracic 1.5–4.0 MHz and with a continuously traced ECG.

### 2.3. Treatment Algorithm

**Figure 1:**
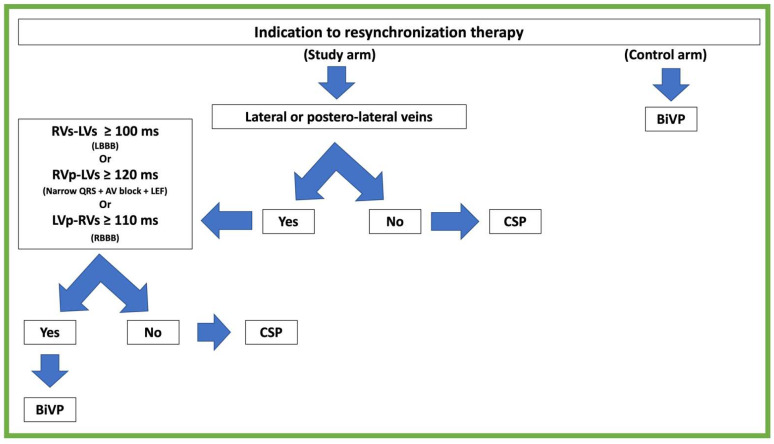
Detailed treatment algorithm. RVs-LVs: interventricular interval between right-ventricle sensed electrogram and left-ventricle sensed electrogram. RVp-LVs: interventricular interval between right-ventricle paced electrogram and left-ventricle sensed electrogram; LVp-RVs: interventricular interval between left-ventricle paced electrogram and right-ventricle sensed electrogram; LEF: low ejection fraction; AV: atrioventricular.

After the positioning of right ventricular (RV) and atrial leads, a quadripolar LV lead was delivered using a Josephson curved catheter into the coronary sinus (CS) guided with CS venography. If the lateral or posterior-lateral vein was accessible, the time interval between the sensed RV and sensed LV (RVs-LVs) was measured during spontaneous rhythm using an LV-pacing quadripolar LV catheter. The recorded RV-LV interval was the longest one measured between the RV tip and any of the various dipole options of the LV lead without phrenic nerve capture (PNC) or high threshold capture value. In all DRG subjects, a quadripolar lead was used to perform BiVP. If the RVs-LVs interval was equal to or longer than a pre-definite interval of (≥) 100 ms, the lead was left in its original position; otherwise, the conduction delay was measured during right ventricular pacing. In cases of LBBB, if the paced RV to sensed LV interval (RVp-LVs) was equal to or longer than a pre-definite interval of (≥) 120 ms, the lead was left in the original position. RV-LV interval cutoffs were pre-defined according to the previous literature and our local experience [12]. Furthermore, the RVp-LVs interval was considered as a surrogate of interventricular conduction disturbance in cases of AVB associated with a reduced left ventricular ejection fraction, because of frequent RVs-LVs < 100 ms in patients with this indication for CRT (these patients usually have a narrow QRS). Similarly, in patients with RBBB, a paced LV to sensed RV (LVp-RVs) interval was considered, and in case of LVp-RVs ≥ 100 ms, the lead was left in the original position. If the lateral or posterolateral vein was inaccessible, or measured IVCD were below previously outlined cutoff values, patients underwent CSP after withdrawal of the LV lead. In case of permanent PNC stimulation at 2× the ventricular capture threshold, other veins first (as long as in lateral or posterior-lateral position) or CSP after were preferred according to the treatment algorithm (see Figure 1). The CRT device programming was performed under direct ECG visualization.

### 2.4. Conduction System Pacing

The criteria to perform CSP in our study design were previously reported (see Figure 1). The procedures were performed by two experienced surgeons (AS and CB) who were trained to perform CSP in 2017. His bundle pacing (HBP) was the first attempted method to obtain CSP. His bundle pacing (HBP) was performed using the Select Secure pacing lead (model 3830, 69 cm, Medtronic) delivered through a fixed (model C315 HIS, Medtronic) or steerable curve sheath (model C304 HIS, Medtronic). First, a quadripolar diagnostic catheter was used to identify and target the His bundle region. Therefore, both the steerable/fixed catheter and lead were introduced into the right ventricle as close as possible to the distal pole of the quadripolar diagnostic catheter. Subsequently, the lead was electrically connected to a polygraph system to analyze the unipolar signals to evaluate the presence of a His bundle signal. His bundle capture was assessed during pace testing and the lead was screwed in in case of selective or nonselective His bundle capture. Threshold and sensing tests were repeated to confirm acceptable electrical parameters (capture threshold < 2 V @ 1 ms). Selective or nonselective His bundle capture was defined according to the criteria described by consensus of a collaborative working group [13]. Left bundle branch pacing (LBBP) was performed as a second option in case of high threshold (>2 V @ 1 ms) with HBP. LBBP was performed according to the previous description and capture was confirmed according to the literature criteria [14,15]. A quadripolar catheter was used to identify the left bundle branch electrogram. Then, the lead was screwed in until the transition from paced LBBB pattern to paced RBBB pattern morphology, and when obtaining the shortest interval between stimulus and peak R wave in V6 with low output. LBBP was defined as the capture of either the proximal left bundle or the septal left bundle with intermediate QRS axis (DII/DIII discordance), or the anterior or posterior fascicles with inferior (both DII and DIII positive) or superior (both DII and DIII negative) QRS axis, respectively.

### 2.5. Endpoints and Clinical Follow-Up

The main endpoint was a composite of cardiovascular death, HF hospitalizations, and urgent unplanned clinic visits because of worsening HF symptoms or fluid status alarm from device telemonitoring at 1 year after the date of intervention. Secondary endpoints were echocardiographic response to CRT and improvement in NYHA class at the 1-year follow-up. ECG data were collected at the 1-year follow-up as well. A positive echocardiographic response to CRT was defined as a reduction of the left ventricular end-systolic volume ≥ 15%. Endpoints were prospectively collected in a dedicated database.

### 2.6. Statistical Analysis

Continuous data were reported as mean ± standard deviation, as appropriate. The Kolmogorov–Smirnov test was used to verify normal distribution of variables. Categorical variables were reported as numbers and percentages. Wilcoxon’s test, ANOVA, and Fisher’s exact or chi-square test were used to test differences between variables, as appropriate. A covariance correlation analysis was performed using Pearson’s test to evaluate the relationship between continuous variables. A multiple stepwise linear regression analysis was conducted to determine the increase of the EF after CRT (ΔEF = EF after CRT-EF before CRT) and NYHA class at the 1-year follow-up. The beta value (b) was the regression coefficient for stepwise multiple linear regression; the b coefficient indicated how the dependent variable responded to changes in the independent variable after adjusting for all other covariates in the model. Time-to-first event analyses were described using Kaplan–Meier estimates and compared between the two groups with a log-rank test. A receiver operating characteristic (ROC) curve was also generated to evaluate the predictive performance of the RVs-LVs interval for echocardiographic response to CRT. The statistical analysis was conducted using the SPSS Statistics 20 (SPSS Inc., Chicago, IL, USA) software package.

## 3. Results

### 3.1. Study Population

Among the considered patients, 25 were excluded from further analysis in the DRG arm, while 17 were excluded from further analysis in the SRG arm. The reasons for exclusion in the DRG were: AF with VP <95% (9 patients), frequent PVC with VP < 95% (10 patients), LV lead dislodgment (3 patients), and follow-up not available (3 patients). The reasons for exclusion in the SRG group were: AF with VP < 95% (10 patients), frequent PVC with VP < 95% (4 patients), and LV lead dislodgment (3 patients). The final study population consisted of 292 patients (mean age 70.2 ± 12.2 years; 28.8% females), of which 160 (54.8%) were in the DRG and 132 (45.2%) in the SRG. Baseline characteristics did not significantly differ between the two groups (see Table 1).

### 3.2. Procedural Aspects

In the DRG, 41 of 160 patients (25.6%) underwent CSP, while in 119 of 160 patients (74.4%), a BiVP was successfully obtained. CSP was performed in 10 patients (24.4%) due to inadequate CS vein anatomy (no lateral or posterior-lateral veins), in 3 patients (7.3%) due to the presence of PNC, and in 28 patients (68.3%) due to inadequate RV-LV intervals, of which 11 patients had LBBB, 9 patients had RBBB, and 8 patients had AVB associated with EF ≤ 45%. CSP was obtained with HBP in 28 of 41 patients (68.3%), either selective in 23 patients (82.1%) or nonselective in 5 patients (17.9%), and LBBP in 13 of 41 patients (31.7%). With regard to BiVP, all LV leads were implanted in a lateral or posterior-lateral branch of the CS. In particular, 15 of 119 patients (12.6%) had the LV lead placed in the basal position, while 104 of 119 patients (87.4%) in a midposition. In the SRG, 107 of 132 patients (81.1%) had the LV lead in the midlateral or posterior-lateral vein of the CS, while 25 of 132 patients (18.9%) had it in the basal-lateral or posterior-lateral vein of the CS.

### 3.3. Procedural Results

**Figure 2:**
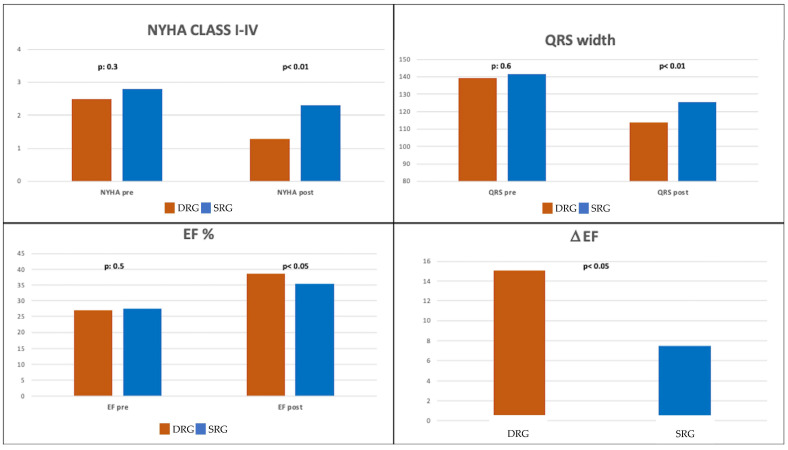
NYHA class, QRS width, EF, and increase of the EF before and after CRT/CSP therapy divided by DRG and SRG. DRG: resynchronization delayed guide group; SRG: resynchronization standard guide group. CSP: conduction system pacing; BiVP: biventricular pacing.

The post-resynchronization QRS width in the DRG was significantly shorter than in the SRG (113.9 ± 13.9 ms vs. 125.4 ± 11.5 ms, respectively, *p* < 0.01). The EF in the DRG was higher than in the SRG (38.8 ± 10.1% vs. 35.4 ± 12.3%, respectively, *p* < 0.05). Both the EDV index (87.9 ± 32.7 mL/m^2^ vs. 92.4 ± 5.7 mL/m^2^) and ESV index (50.5 ± 29.2 mL/m^2^ vs. 56.1 ± 31.4 mL/m^2^) were lower in the DRG group compared to the SRG group, respectively (*p* < 0.05). The mean post-resynchronization NYHA class was lower in the DRG compared to the SRG (1.3 ± 0.5 vs. 2.3 ± 0.6, respectively; *p* < 0.01). For procedural results, see Table 2 and Figure 2.

### 3.4. Endpoints

**Figure 3:**
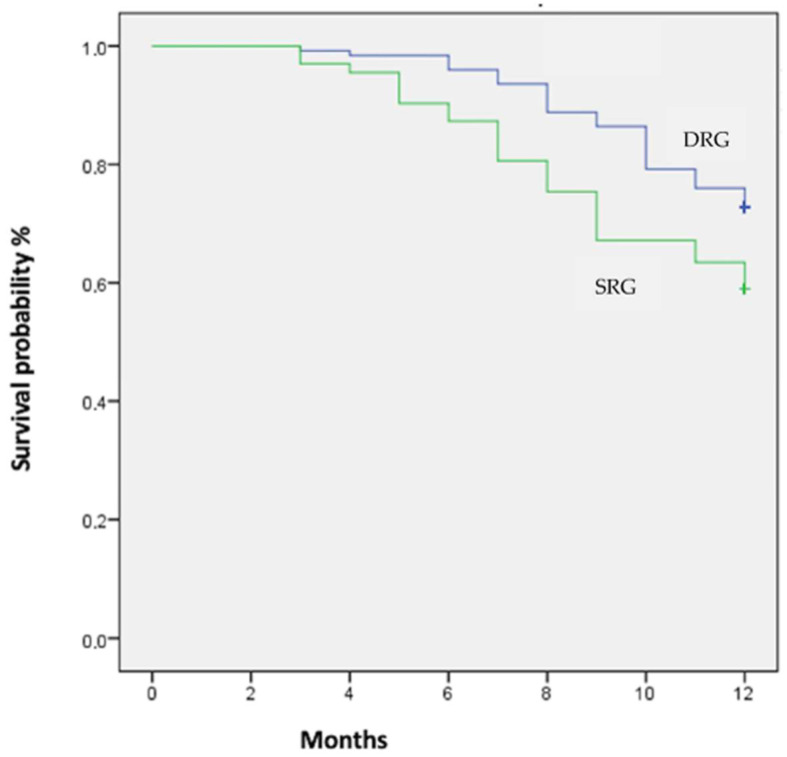
Kaplan–Meier curves for primary outcome at the 1-year follow-up stratified for DRG and SRG.

There was a significant difference in the incidence of the primary endpoint between the two groups, with more events in the SRG compared to DRG, as reported in Table 3 (hazard ratio (HR): 1.72; 95% confidence interval (CI): 1.12–2.65; *p* = 0.013). For Kaplan–Meier curves, see Figure 3. The observed difference in the occurrence of the primary endpoint appeared to be mainly driven by the difference in the incidence of HF hospitalizations and urgent clinic visits between the two groups (both *p* < 0.01). With regard to secondary outcomes: (a) the percentage of echocardiographic responders in the DRG was greater compared to the SRG (77.5% vs. 63.6%; *p* < 0.01); (b) the mean post-resynchronization NYHA class was lower in the DRG than in the SRG (1.3 ± 0.5 vs. 2.3 ± 0.6; *p* < 0.01). On multivariate analysis, only DRG was predictive of improvement of the NYHA class after CRT (HR: 0.6, 95% CI: 0.3–0.9, *p* < 0.01), while the following baseline features were not predictive, including: LBBB or RBBB, NYHA class, BSA sex, QRS width, and HF etiology. See Table 4 for results of multivariate analysis. Multivariate analysis also confirmed DRG as an independent predictor of EF improvement (HR = 0.5, 95% CI: 0.3–0.9, *p* < 0.01), along with non-ischemic HF etiology (HR = 0.5, 95% CI= 0.2–0.9, *p* < 0.01).

### 3.5. Interventricular Conduction Delays

**Figure 4:**
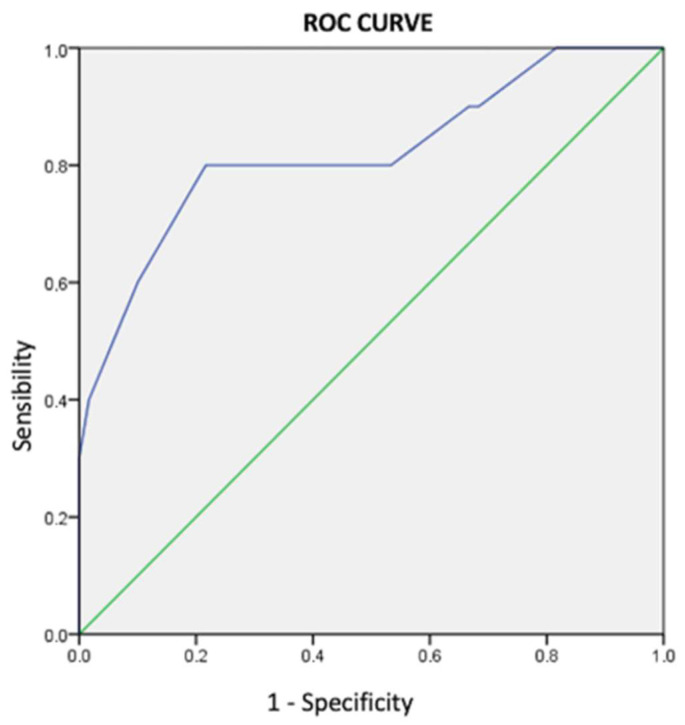
Receiver operating characteristic curve for prediction of echocardiographic response to CRT/CSP by RVs-LVs interval.

The RVs-LVs interval showed a weak linear correlation with the RVp-LVs interval (r: 0.4; *p* < 0.01) and LVp-RVs interval (r: 0.4; *p* < 0.01). Finally, the ROC curve confirmed the RVs-LVs cutoff value of 100 ms to be a reliable threshold to identify when to use CSP versus BiVP during CRT (RDG strategy) versus conventional empiric CRT (SRG strategy) (AUC = 0.821, 95% CI: 0.655–0.986; *p* < 0.001) (Figure 4).

## 4. Discussion

The main finding of this study is that the application of a treatment algorithm based on IVCD for patients with an indication for CRT reduced the incidence of the primary outcome, a composite of cardiovascular deaths, HF hospitalizations, and urgent unplanned clinic visits at the 1-year follow-up.

The typically examined parameters to evaluate the potential benefit of CRT are female sex, BSA, QRS width and morphology, and HF etiology [16]. Nonetheless, despite accounting for these factors and the use of quadripolar leads, lateral or posterior-lateral vein positioning, and pacing optimization, the number of non-responders to CRT is still high, ranging between 30% and 40% [17,18]. The intrinsic interventricular conduction interval, easily measured with surrogates such as spontaneous or paced interventricular intervals after LV lead positioning, could better predict the clinical benefit of CRT with BiVP [17,19].

In this study, we applied a treatment algorithm based on IVCD for patients with an indication for CRT. Particularly, spontaneous and paced interventricular intervals were used to decide whether to perform CRT with BiVP or shift to CSP. This study demonstrated better results in terms of QRS width after CRT/CSP, reverse remodeling, and improvement in functional class in the DRG compared to SRG, which eventually translated in a reduction in the primary endpoint, a composite of cardiovascular deaths, HF hospitalizations, and urgent unplanned clinic visits.

The RVs-LVs interval proved to be a predictor of echocardiographic response to CRT, and a cutoff of 100 ms was both sensible and specific. This finding further supports the use of an RVs-LVs interval with a cutoff of 100 ms in our treatment algorithm to decide between BiVP and CSP. In case of indication for CRT with narrow QRS, namely for AVB with associated reduced EF, the RVp-LVs interval was used as a surrogate of spontaneous interval. Of note, in case of a short RVp-LVs interval, CSP was preferred either because of suboptimal LV lead positioning or because of the absence of an interventricular conduction defect. In case of RBBB, the LVp-RVs interval was used as a surrogate of spontaneous interval, to provide specular criteria with regard to LBBB and RVp-LVs interval use in case of a normal RVs-LVs interval. The use of a paced interval was a fundamental item of the treatment algorithm and, along with spontaneous intervals, predicted CRT response differently from the previous report [19].

Moubarak et al. showed that intrinsic and RV-paced interventricular electrical intervals, although correlated, are not always equivalent in terms of propagation of activation [12]. However, LV leads were positioned in anterior, anterolateral, lateral, and posterolateral veins, whereas in our study, LV leads were positioned only in lateral or posterior-lateral veins. The direction of electrical activation and the latest-activated LV electrode in RVs-LVs and RVp-LVs were dissimilar in 47% and 18% of patients, respectively, and this has potentially important implications in choosing the LV pacing vector. Both RV and LV pacing prolong the interventricular interval compared to intrinsic bundle branch block, but to a different extent, and with substantial heterogeneity between patients [12]. Further studies are needed to determine the clinical significance and applications of RV-LV intervals. In particular, the RVp-LVs interval could help in selecting the optimal LV vector and pacing modality (i.e., biventricular or LV-only pacing).

In conclusion, our study demonstrates that long interventricular conduction intervals are associated with improvement in systolic function and NYHA class after CRT with conventional BiVP. Specifically, the use of the RVs-LVs interval to determine when to perform CSP in patients with an indication for CRT resulted in a significant increase in the number of responders compared to the SRG.

### 4.1. Clinical Implications

Despite being suggested as a valuable alternative to standard CRT, there is no clear consensus on when CSP should be performed to obtain a successful resynchronization yet. Current guidelines only recommend CSP in case of unsuccessful coronary sinus lead implantation in lieu of BiVP, or in case of AVB with associated reduced EF in lieu of RV pacing, without clear definition on when to choose it instead of BiVP. The results of this study suggest that the analysis of the RVs-LVs interval could be a valuable strategy to identify the need for CSP. In the presence of an interventricular conduction interval < 100 ms, performing CSP improved resynchronization when compared to patients with a similar conduction interval but treated with BiVP. The hybrid CSP/BiVP approach, driven by the analysis of conduction intervals, reduced the primary endpoint and increased the number of responders. This suggests that electrical resynchronization should be considered to obtain successful mechanical resynchronization, reverse modeling, and improvement in the NYHA class.

### 4.2. Study Limitations

The main limitation of this study is represented by the small number of enrolled patients and, in particular, those who underwent CSP. Due to the low number, it was not possible to perform further subgroup analysis of patients according to the baseline QRS width, or to directly compare outcomes of patients who received BiVP versus CSP. However, a direct comparison between outcomes of BiVP and CSP was not the scope of this investigation. Another important limitation is the nonrandomized study design. Hence, larger, randomized studies are needed to confirm the results of this pilot study and to assess the efficacy of this hybrid approach to improve patients’ outcomes. A third limitation is the inclusion of patients with different baseline conduction disorders (LBBB, RBBB, and AVB associated with reduced EF) that made the interpretation of the results less specific for the different cohorts of patients to resynchronize. The fourth limitation is represented by the technique used to position the CSP lead monitoring the His signals through fluoroscopy and polygraph. The use of an electroanatomical mapping system is thus suggested for further investigation to reduce the fluoroscopic exposure both for patients and medical staff during the CSP approach. Finally, the last limitation of the study is that QLV (the time interval between the onset of the QRS complex at surface ECG and the intracardiac signal at LV lead) was not reported in addition to the other measures of IVCD. However, according to a recent study, only RV-LV intervals, not QLV, are associated with CRT response [20]. In addition, manual measurement is required for QLV calculation, therefore adding potential error, while many commercially available devices have algorithms to measure interventricular conduction delays automatically.

## 5. Conclusions

The proposed strategy for resynchronization based on the hybrid CSP/BiVP approach according to the RVs-LVs conduction delay represents a feasible and promising solution to increase the number of CRT responders and reduce the risk of cardiovascular deaths, HF hospitalizations, or urgent clinic visits at follow-up.

## Figures and Tables

**Table 1:** Baseline characteristics of the study population stratified for DRG and SRG.

Baseline Characteristics	DRG (*n* = 160)	SRG (*n* = 132)	*p*-Values
***Demographic, Anthropomorphic, Clinical***			
Age (years)	69.9 ± 10.6	70.6 ± 12.7	0.80
Female (n/%)	44/27.5	40/30.0	0.30
BSA (m^2^)	1.82 ± 0.20	1.79 ± 1.20	0.08
Ischemic etiology (n/%)	75/46.8	49/37.1	0.20
History of AF (n/%)	30/18.7	28/21.2	0.10
NYHA class	2.5 ± 1.9	2.8 ± 1.3	0.30
***Electrocardiogram***			
QRS width (ms)	139 ± 22	141 ± 30	0.60
LBBB (n/%)	120/75.1	111/84.1	0.10
AVB (with rEF) (n/%)	14/8.7	16/12.1	0.09
RBBB (n/%)	11/6.8	5/3.8	0.20
***Echocardiogram***			
EF (%)	26.9 ± 9.1	27.5 ± 6.9	0.50
EDD (mm)	67.1 ± 10.5	70.0 ± 3.5	0.07
ESD (mm)	48.6 ± 9.3	50.0 ± 5.3	0.10
EDD index (mm/m^2^)	36.8 ± 3.2	39.1 ± 2.7	0.09
ESD index (mm/m^2^)	26.7 ± 4.1	27.8 ± 1.1	0.30
EDV (mL)	172.4 ± 49.1	174.4 ± 34.2	0.20
ESV (mL)	120.6 ± 46.2	123.2 ± 41.0	0.09
EDV index (mL/m^2^)	94.5 ± 26.9	97.2 ± 18.9	0.07
ESV index (mL/m^2^)	65.9 ± 25.3	68.7 ± 22.9	0.09
***Medical therapy***			
Beta-blockers (n/%)	143/89.3	111/84.1	0.30
ACEI-ATIIra-Sa/Va (n/%)	149/ 93.1	123/93.2	0.90
Diuretics (n/%)	115/71.8	111/84.1	0.09
Aldosterone antagonist (n/%)	75/46.8	53/40.2	0.40

DRG: resynchronization delay guided group; SRG: resynchronization standard group; BSA: body surface area; EF: ejection fraction; LBBB: left bundle branch block; AVB: atrioventricular block; rEF: reduced ejection fraction: EF ≤ 45%. RBBB: right bundle branch block; AF: atrial fibrillation; EDD: end-diastolic diameters; ESD: end-systolic diameters; EDD index: end-diastolic diameters indexed by BSA; end-systolic diameters indexed by BSA; EDV: end-diastolic volume; ESV: end-systolic volume; EDV index: end-diastolic volume indexed by BSA; ESV index: end-systolic volume indexed by BSA; ACEI: Ace inhibitors; ATIIra: angiotensin II receptor antagonist; Sa/Va: sacubitril/valsartan.

**Table 2:** Procedural results after CRT stratified for DRG and SRG.

Procedural Results	DRG (*n* = 160)	SRG (*n* = 132)	*p*-Values
***Clinical***			
NYHA class	1.3 ± 0.5	2.3 ± 0.6	<0.05
***Electrocardiogram***			
QRS width (ms)	113 ± 13	125 ± 25	<0.01
BiVP or CSP stimulation (%)	98.2 ± 10.1	97.7 ± 1.8	0.80
CSP stimulation (*n*/%)	41/25.6	0/0	-
HBP (*n*%)	28/68.3	0/0	
sHBP (*n*.)	23	0	
nsHBP (*n*.)	5	0	
LBBP (*n*/%)	13/31.7	0/0	
***Echocardiogram***			
EF (%)	38.8 ± 10.1	35.4 ± 12.3	<0.05
EDD (mm)	57.4 ± 9.8	62.3 ± 8.3	<0.05
ESD (mm)	45.7 ± 11.7	49.1 ± 10.3	<0.05
EDD index (mm/m^2^)	31.5 ± 5.4	34.8 ± 4.6	<0.05
ESD index (mm/m^2^)	25.1 ± 5.3	28.4 ± 5.8	0.10
EDV (mL)	160.1 ± 59.6	165.4 ± 8.9	0.09
ESV (mL)	90.1 ± 53.1	99.7 ± 52.2	<0.05
EDV index (mL/m^2^)	87.9 ± 32.7	92.4 ± 5.7	<0.05
ESV index (mL/m^2^)	50.5 ± 29.2	56.1 ± 31.4	<0.05
ΔEF (%)	11.3 ± 10.5	7.5 ± 13.7	<0.01

EF: ejection function; CSP: conduction system pacing; HBP: His bundle pacing; sHBP: selective His bundle pacing; nsHBP: nonselective His bundle pacing; LBBP: left bundle branch pacing; EDD: end-diastolic diameter; ESD: end-systolic diameter; EDD index: end-diastolic diameter indexed by BSA; ESD index: end-systolic diameter indexed by BSA; EDV: end-diastolic volume; ESV: end-systolic volume; EDV index: end-diastolic volume indexed by BSA; ESV index: end-systolic volume indexed by BSA; ΔEF: delta ejection fraction (EF after CRT/CSP–EF before CRT/CSP).

**Table 3:** Primary (composite outcome) and secondary (echocardiographic response and NYHA class) endpoints stratified for DRG and SRG.

Endpoints	DRG (*n* = 160)	SRG (*n* = 132)	*p*-Values
Composite outcome	52 (32.5)	74 (56.0)	<0.01
Cardiovascular death (*n*/%)	11 (6.9)	16 (12.1)	0.09
HF hospitalization (*n*/%)	15 (9.4)	43 (32.6)	<0.01
Urgent clinic visit (*n*/%)	29 (18.1)	50 (37.9)	<0.01
Echocardiographic responders (*n*/%)	124 (77.5)	84 (63.6)	<0.01
NYHA class	1.3 ± 0.5	2.3 ± 0.6	<0.05

**Table 4:** Results of multivariate analysis for the improvement in NYHA class and EF. DRG: resynchronization delayed guide group. ΔEF: delta ejection fraction (EF after CRT–EF before CRT).

Determinants of NYHA Class Improvement			
Parameter	B	HR (95% CI)	*p*-Values
DRG	0.3	0.6 (0.3–0.9)	0.001
**Determinants of** **ΔEF**			
**Parameter**	**B**	**HR (95% CI)**	***p*-Values**
DRG	0.2	0.5 (0.3–0.9)	0.010
HF etiology	0.2	0.5 (0.2–0.9)	0.010

## Data Availability

Not applicable.

## Abbreviations

CRT, cardiac resynchronization therapy; CSP, conduction system pacing; IVCD, interventricular conduction delays; BiVP, biventricular pacing; HBP, His bundle pacing; LBBP, left bundle branch pacing; LV, left ventricular; LBBB, left bundle branch block; RBBB, right bundle branch block; HF, heart failure; DRG, delays-guided resynchronization group; SRG, resynchronization standard guide group; AV, atrioventricular; BSA, body surface area; OMT, optimal medical therapy; AVB, atrioventricular block; AF, atrial fibrillation; VP, ventricular pacing; PVC, premature ventricular complex; RV, right ventricular; CS, coronary sinus; PNC, phrenic nerve capture; EF, ejection fraction.
